# Sex differences in glutamate AMPA receptor subunits mRNA with fast gating kinetics in the mouse cochlea

**DOI:** 10.3389/fnsys.2023.1100505

**Published:** 2023-03-02

**Authors:** Nicholas R. Lozier, Steven Muscio, Indra Pal, Hou-Ming Cai, María E. Rubio

**Affiliations:** ^1^Department of Neurobiology, University of Pittsburgh, Pittsburgh, PA, United States; ^2^Department of Otolaryngology, University of Pittsburgh, Pittsburgh, PA, United States; ^3^Center for the Neural Basis of Cognition, University of Pittsburgh, Pittsburgh, PA, United States

**Keywords:** spiral ganglion neurons (SGNs), gene expression, auditory brainstem response (ABR), inner ear, qRT-PCR, splice variants AMPA receptors

## Abstract

Evidence shows that females have increased supra-threshold peripheral auditory processing compared to males. This is indicated by larger auditory brainstem responses (ABR) wave I amplitude, which measures afferent spiral ganglion neuron (SGN)-auditory nerve synchrony. However, the underlying molecular mechanisms of this sex difference are mostly unknown. We sought to elucidate sex differences in ABR wave I amplitude by examining molecular markers known to affect synaptic transmission kinetics. Alpha-amino-3-hydroxy-5-methyl-4-isoxazolepropionic acid receptors (AMPARs) mediate fast excitatory transmission in mature SGN afferent synapses. Each AMPAR channel is a tetramer composed of GluA2, 3, and 4 subunits (*Gria2, 3*, and *4* genes), and those lacking GluA2 subunits have larger currents, are calcium-permeable, and have faster gating kinetics. Moreover, alternatively spliced *flip* and *flop* isoforms of each AMPAR subunit affect channel kinetics, having faster kinetics those AMPARs containing *Gria3* and *Gria4 flop* isoforms. We hypothesized that SGNs of females have more fast-gating AMPAR subunit mRNA than males, which could contribute to more temporally precise synaptic transmission and increased SGN synchrony. Our data show that the index of *Gria3* relative to *Gria2* transcripts on SGN was higher in females than males (females: 48%; males: 43%), suggesting that females have more SGNs with higher *Gria3* mRNA relative to *Gria2*. Analysis of the relative abundance of the *flip* and *flop* alternatively spliced isoforms showed that females have a 2-fold increase in fast-gating *Gria3* *flop* mRNA, while males have more slow-gating (2.5-fold) of the *flip*. We propose that *Gria3* may in part mediate greater SGN synchrony in females.

**Significance Statement:** Females of multiple vertebrate species, including fish and mammals, have been reported to have enhanced sound-evoked synchrony of afferents in the auditory nerve. However, the underlying molecular mediators of this physiologic sex difference are unknown. Elucidating potential molecular mechanisms related to sex differences in auditory processing is important for maintaining healthy ears and developing potential treatments for hearing loss in both sexes. This study found that females have a 2-fold increase in *Gria3 flop* mRNA, a fast-gating AMPA-type glutamate receptor subunit. This difference may contribute to greater neural synchrony in the auditory nerve of female mice compared to males, and this sex difference may be conserved in all vertebrates.

## Introduction

Females of multiple mammalian species, including mice, rats, and humans, are better protected from sensorineural hearing loss (SNHL). Females also have enhanced supra-threshold peripheral auditory processing independent of hearing loss (Balogová et al., 2018; Milon et al., 2018; for review, see Lin et al., 2022) which has been recorded using auditory brainstem responses (ABRs). Indeed, females were found to have higher wave I amplitudes in mice (Milon et al., 2018), rats (Balogová et al., 2018), and humans (Kjær, 1979, 1980; Wharton and Church, 1990). Most of these previous studies show that females have higher amplitudes in later waves (i.e., higher-order auditory nuclei), thus the cochlear nerve is not the only location in the auditory processing pathway for this sex difference. Still, our current study focuses on potential sex effects at the level of the auditory nerve (wave I). ABR wave I is a measure of the response of multiple afferent fibers in the auditory nerve to sound, so larger ABR wave I amplitude suggests that females have greater synchrony of spiral ganglion neuron (SGN) afferent fibers or recruitment of more SGNs of a specific population at higher sound levels (Young et al., 2021). While sex hormones including estradiol and testosterone modulate auditory afferent synchrony in fish (Sisneros et al., 2004) and mice (Shuster et al., 2021), the underlying molecular mechanism of the sex difference in peripheral auditory processing in healthy inner ears of female and male mammals remains almost entirely unexplored. Understanding how healthy peripheral auditory systems differ between males and females is essential for tailoring specific hearing loss treatments and maintaining optimal auditory processing in both biological sexes.

Fast excitatory transmission of information about sound between cochlear inner hair cells and SGN afferents is mediated by α-amino-3-hydroxy-5-methyl-4-isoxazole propionic acid receptors (AMPARs; Puel et al., 1991; Ruel et al., 1999, 2000; Glowatzki and Fuchs, 2002; Sebe et al., 2017). The larger wave I amplitude in females could result, at least in part, from SGNs that are more synchronous due to more temporally precise AMPAR-driven excitatory postsynaptic currents (EPSCs). AMPARs are tetramers assembled as dimers of dimers (Rosenmund et al., 1998; Tichelaar et al., 2004). In the mature cochlea, SGNs express GluA2, GluA3, and GluA4 subunits (Niedzielski and Wenthold, 1995; Matsubara et al., 1996). AMPARs can affect the speed of postsynaptic depolarization by changes in overall expression level that affect current density, or by changes in subunit relative abundance that affect channel permeability and gating kinetics (Trussell, 1997, 1999). AMPAR subunits influence channel permeability to sodium and calcium, and the gating kinetics of the channel is determined by the *flip-and-flop* splice variants (Mosbacher et al., 1994). Each AMPAR subunit occurs in two possible isoforms, *flip* and *flop*, depending on the splicing of a segment in the mRNA sequence that encodes the protein region just before the fourth transmembrane domain (Sommer et al., 1990; Dingledine et al., 1999). Subunits of the alternatively spliced *flop* isoform have faster gating kinetics than the *flip* isoform, especially *Gria3* and *Gria4* (Mosbacher et al., 1994). We hypothesized that sex differences in SGN synchrony result, at least in part, from differences in the speed and reliability of synaptic transmission due to differences in the expression of AMPAR subunits and their splice variants in females and males.

This study sought to determine whether differential expression of AMPAR subunits could contribute to the underlying sex difference in ABR wave I amplitude, indicating higher SGN synchrony in C57BL/6J female mice. To test our hypothesis, we used *in situ* hybridization to quantify *Gria2*, *3*, and *4* mRNA levels in type I SGNs from the basal, middle, and apical cochlear regions. In addition, we quantified the relative abundances of *flip* and *flop* mRNA isoforms for each subunit with RT-qPCR. Our study shows that females have larger ABR wave I amplitude, confirming this sex difference in C57BL/6J mice. Females also had more overall AMPAR mRNA, in particular *Gria3 flop*. We conclude that of the three AMPAR subunits in the mature cochlea, *Gria3* has the greatest potential to mediate the difference in wave I amplitude between females and males.

## Materials and methods

### Animals

A total of eighty-five C57BL/6J mice were used in this study. Auditory brainstem response (ABR) experiments were conducted on a cohort of mice with an average age of postnatal day 37 (P37, range = 34–40; female *n* = 17, male *n* = 13), and a cohort with an average age of P60 (range = P57–P73; female *n* = 10, male *n* = 13). Throughout the remainder of the study, these mice will be referred to as the P37 and P60 cohorts, respectively. For *in situ* hybridization (female *n* = 4, male *n* = 4) and RT-qPCR (female *n* = 12, male *n* = 12), mice were aged P34–50. C57BL/6J mice in these age ranges are sexually mature (Bell, 2018). ABR data were collected on two sexually mature age cohorts to ensure that the sex difference in ABR wave I amplitude was maintained in the age ranges used for *in situ* hybridization and RT-qPCR. C57BL/6J mice were used in this study because they are the most commonly used strain for genetic modifications. We wanted to establish this sex difference in WT C57BL/6J mice for potential future experiments examining the mechanisms of this sex difference in genetically modified mice. The age range of the C57BL/6J mice used in this study is before the age where early-onset age-related hearing loss has been previously documented in this strain, which was P100 for high frequencies beyond the age range used in this study (Henry, 2002), and P150 for a broadband threshold shift (Henry, 2004). Mice were housed in a room with ambient noise levels averaging 15–20 dB SPL (unweighted mean SPL, 3 kHz–90 kHz, Sensory Sentinel, Turner Scientific), and a 12-h light/12-h dark daily photoperiod. Mice were fed *ad libitum*. All procedures were carried out in accordance with the National Institute of Health Guide for the Care and Use of Laboratory Animals and were approved by the University of Pittsburgh Institutional Animal Care and Use Committee.

### Auditory brainstem responses

Auditory brainstem responses (ABRs) were recorded and analyzed in a similar protocol to that used by Clarkson et al. (2016) and García-Hernández et al. (2017). However, the sound was presented in the open field in this study. Recordings were done in a Faraday cage in a sound attenuation chamber. During ABR experiments, mice were continuously anesthetized with 1.5% isoflurane in oxygen, and body temperature was kept constant *via* isothermal heat pads. The hydration and internal temperature of the mice were not measured; however isothermal heat pads were heated under similar conditions for each experiment. Thus, the external environment was controlled between mice. The speaker was calibrated in the chamber using a microphone (PCB electronics, model no. 377C01, Depew, NY) placed 10 cm from the multi-field magnetic speaker (MF1, Tucker-Davis Technologies (TDT), Alachua, FL), which was the same distance as the mouse’s external ear to the speaker during experiments. Broadband click and pure tone burst stimuli (4, 8, 12, 16, 24, and 32 kHz) were presented in the open field. For clicks, stimuli were presented for 0.1 ms starting at 90 dB and decreased to 10 dB in 5 dB steps, while pure tone stimuli were presented for 5 ms (2.5 ms linear rise/fall time with no plateau) at 90 dB and decreased to 20 dB in 10 dB steps. All stimuli were presented at 21 sweeps/s (i.e., stimulus period = 47.619 ms). Subdermal electrodes were placed on the vertex of the skull (recording electrode), ipsilateral (right) ear mastoid through the cheek (reference electrode), and contralateral (left) ear mastoid through the cheek (ground electrode). All ABRs were recorded in 10 ms increments. Incoming signals were digitized and recorded using a RA4LI headstage, RA4PA preamplifier/digitizer, and RZ6 signal processor (TDT). Data were collected in real-time using PC interface BioSigRZ software (TDT). All recordings were averaged over 512 sweeps and were band-pass filtered from 300 to 3,000 Hz. ABR click and pure tone threshold were operationally defined as the lowest stimulus level at which ABR waveforms could be visualized. Wave I latencies and amplitudes were calculated from click ABR recordings. Latency of wave I was defined as the time from the onset of the stimulus to the peak of wave I in ms, and wave I amplitude was defined as the peak of wave I to the subsequent trough in μV.

### *In situ* hybridization

Mice were anesthetized *via* intraperitoneal injection with a mixture of ketamine (60 mg/kg) and xylazine (6.5 mg/kg) and transcardially perfused with 0.1 M phosphate buffer (PB) followed by 4% paraformaldehyde (PFA) in 0.1 M phosphate-buffered saline (PBS) in RNAse-free water. Cochleae were carefully removed and perilymphatically perfused with 4% PFA in 0.1 M PBS and then immersion fixed for 1–3 h on ice. Immediately following immersion-fixation, cochleae were moved to 10% EDTA in 0.1 M PBS and gently agitated overnight at 4°C for decalcification. The next morning, cochleae were washed in 0.1 M PBS, serially dehydrated in 10%, 20%, and 30% sucrose in 0.1 M PBS, and frozen on dry ice in molds with tissue freezing medium (Electron Microscopy Sciences, Hatfield, PA), and stored at −20°C for one month. The right cochlea of each mouse was sectioned at 12 μm thickness using a cryostat. Four sections were adhered per slide in series and stored at −80°C for 6 months prior to *in situ* hybridization.

Each *in situ* hybridization experiment used slides from at least one male and one female. Slides were chosen for each experiment in an attempt to control for equal numbers of SGNs per mouse at each cochlear region. *In situ* hybridization was carried out using probes, amplifiers, and fluorescent dyes from the RNAscope multiplex fluorescent assay developed by ACDBio (Newark, CA). This included probes for *Gria2* (cat. no. 416091-C2), *Gria 3* (cat. no. 426251-C3), and *Gria4* (cat. no.422801-C1) as well as a kit necessary for amplification and labeling steps (RNAscope Multiplex Fluorescent Reagent Kit v2, cat. no. 323100). The probes do not differentiate between the *flip* and *flop* isoforms of each subunit. Probe specificity was validated in each experiment by including a slide that was incubated with a positive control probe mixture (3-Plex positive control probe, cat no. 320881), and a slide that was incubated with a negative control probe mixture (3-Plex negative control probe, cat no. 320871) in each experiment. Due to the limitations of the assay, only two RNA probes were targeted in each experiment. Therefore, in half of the experiments, *Gria2* and *Gria3* were targeted in tandem, while in the other half, *Gria2* and *Gria4* were targeted in tandem. *Gria2* was targeted in every experiment both to serve as a control between experiments and to allow for calculating the index of *Gria3* and *Gria4* relative to *Gria2* mRNA in individual SGNs (see Section “Statistical analysis” below). In all experiments, the *Gria2* probe was labeled with Opal 690 dye, while *Gria3* and *Gria4* were labeled with Opal 570 dye (Akoya Biosciences, Marlborough. MA, cat. no. FP1497001KT and FP1488001KT, respectively). Following hybridization, amplification, and labeling, slides were mounted in ProLong Gold with DAPI (Invitrogen, Eugene, OR, cat. no. P36931), cover-slipped, and stored at 4°C away from light for up to one month prior to confocal imaging.

### Confocal imaging and fluorescence puncta quantification

**Figure 1:**
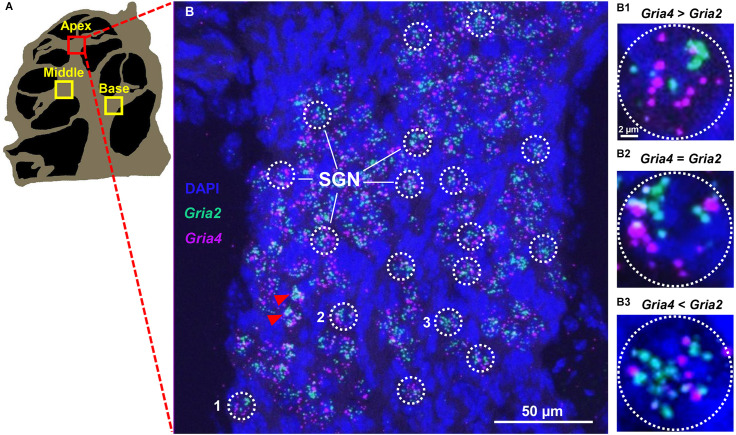
Specific expression of *Gria2* and *Gria4* mRNA puncta on SGN. **(A)** A computer-generated sketch of a cochlear section through the modiolus. The spiral ganglion (indicated with squares) is located on the modiolar side of sensory epithelia and contains a group of neuron cell bodies (the spiral ganglion neurons, SGNs). **(B)** A representative maximum-intensity projection of a confocal stack of images of SGNs of the apical cochlea turn of a male mouse with mRNA signal for *Gria2* (green) and *Gria4* (magenta), and DAPI nuclei staining (blue). The mRNA signal for *Gria2* and *Gria4* is observed as puncta grouped around large DAPI nuclei. ROIs (white dotted line circles) correspond to individual SGNs. Red arrowheads point to SGNs that were excluded due to puncta clustering. **(B1–3)** High magnification of the three SGNs (1, 2, and 3) containing different ratios of *Gria2* and *Gria4* puncta. In **(B1)**, the SGN has more *Gria4* puncta than *Gria2*. In **(B2)**, the SGN has equal amounts of *Gria4* puncta and *Gria2*. In **(B3)**, the SGN has less *Gria4* puncta than *Gria2*. See Section “Methods” and Figure 6 for details of the quantitative analysis.

All slides were imaged with a Nikon A1 confocal inverted stage microscope (Nikon Instruments, Inc.) at the University of Pittsburgh Center for Biologic Imaging. DAPI, Opal 570, and Opal 690 were imaged simultaneously from lasers with excitation/emission spectra filters set at 405/425–475, 561/570–620, and 640/663–738 nm, respectively with a 40× 1.3 NA oil immersion lens and pinhole set to 1 airy unit. Images were collected in z-stacks of 0.25 μm increments using NIS Elements software (Nikon). Image stacks were combined into one z-projection containing all three fluorescence channels using Image-J^1^. Opal 690 (*Gria2*) puncta were pseudo-colored to green, Opal 570 (*Gria3* or *Gria4*) puncta were pseudo-colored to magenta, and DAPI remained blue (see Figure 1). ROIs (approximately 12 μm in diameter) for puncta counts were defined as separate type I-like SGNs, which were identified in each image by the presence of opal 690 (green) and opal 570 (magenta) puncta clustered around large DAPI-stained nuclei (see Figure 1). To ensure that clusters of transcript puncta were segregated to one SGN, we spot-checked multiple z-stacks for each cochlea to ensure that there was no overlap of SGNs in the z-plane. Amounts of *Gria3* or *Gria4* and *Gria2* puncta were manually counted in individual SGNs semi-quantitatively: green and magenta puncta were counted separately using the Image-J point tool. On rare occasions, puncta were observed in large clusters where individual puncta could not be distinguished. Puncta with diameters exceeding 14 pixels were considered clustered, and these SGNs were excluded from the analysis, similar to the methods in Balaram et al. (2019). Puncta with diameters less than 2 pixels were considered noise and were not counted within SGNs. *Gria2* and *Gria3* counts are likely under-reported because these subunits tended to cluster the most. However, only up to four SGNs per image (out of over 30 in most images) were excluded from the analysis, and many of those images spot-checked (18 out of 30) had no clustering. See red arrowheads in Figures 1B, 3A for examples of SGNs that were excluded due to clustering. The intensity of puncta fluorescence was not considered in the analysis. On average, puncta from 30 SGNs were quantified per image. Throughout all experiments, four-six slides containing four cochlear sections each were probed per mouse. All fluorescence analysis was performed by observers blind to the sex of the animal.

### Quantitative reverse transcription-polymerase chain reaction

Mice were anesthetized with isoflurane and euthanized *via* cervical dislocation and decapitation. Immediately following decapitation, the cranium was opened. The inner ears were removed, flash-frozen in liquid nitrogen, and stored at −80°C for up to one month until reverse transcription-polymerase chain reaction (RT-PCR). In preparation for RT-PCR, both inner ears from each mouse were homogenized by hand with mortar and pestle and RNA was extracted with Trizol (Ambion by life technology). The pellet was resuspended and the supernatant containing RNA from each inner ear (including auditory and vestibular tissue) was prepared for RT- PCR using the SuperScript Strand Synthesis System kit (Invitrogen, cat. no. 11904018). The resulting cDNA was stored at −20°C for 1 week or less prior to quantitative PCR (qPCR). qPCR was performed at the Genomics Research Core at the University of Pittsburgh using EvaGreen qPCR kit (MidSci, Valley Park, MO, cat. no. BEQPCR_R) and DNA primers for *Gria2*, *Gria3*, and *Gria4 flip* and *flop*, which were the same primers used successfully in a previous RT-qPCR experiment by Hagino et al. (2004); see Table 1 in this reference for sequence information. In a 25 μl PCR reaction mixture, 2 μl cDNA samples were amplified in a Chromo 4 detector (MJ Research, Waltham, MA). GAPDH and 18S rRNA were used as housekeeping genes. Each sample (consisting of cDNA product of both cochleae from each mouse) was run in triplicate, and average cycle thresholds (CTs) were used for quantification. Relative abundances of each splice isoform for males compared to females were calculated using the following equation: 2^−ΔΔCT^, where ^ΔΔCT^ = (CT_Male_ − CT_GAPDH_ or CT_18S rRNA_) − (CT_Female_ − CT_GAPDH_ or CT_18S rRNA_). For a more in-depth explanation of this equation see Schmittgen and Livak (2008).

### Statistical analyses

All statistical tests were calculated using GraphPad Prism or IBM SPSS software. ABR thresholds, wave I latency and wave I amplitude were compared between males and females using separate two-way mixed ANOVAs for P37 and P60 mice. For comparisons of click wave I latency and wave I amplitude, the within-subjects independent variable (IV) was sound level (90–50 dB in 5 dB increments). In contrast, for ABR thresholds the within-subjects IV was sound frequency (click, 4, 8, 12, 16, 24, and 32 kHz). For these two-way mixed ANOVAs, the between-subjects IV was sex. For latency and amplitude, linear regressions were calculated to compare the slope of ABR wave I latency or amplitude as a function of sound level between males and females of P37 and P60. For wave I amplitude slopes, Tukey pairwise comparisons were calculated between each group.

For RNAscope mRNA quantification data, three separate two-way ANOVAs were conducted to compare *Gria2*, *3*, and *4* mRNA quantities between males and females (IV1, sex) in the basal, middle, and apical cochlear turns (IV2, region). To quantify possible differences in the expression of *Gria2* vs. *Gria3* and *Gria2* vs. *Gria4* in individual SGNs, we calculated a *Gria3/2* and *Gria4/2* index with the following equation, derived from Balaram et al. (2019): (*Gria3* or *Gria4* − *Gria2*)/(*Gria3* or *Gria4* + *Gria2*). A positive-value index means that a SGN has more *Gria3* or *Gria4* relative to *Gria2*; a value of zero means that *Gria3* or *Gria4* and *Gria2* puncta levels in a SGN are equal; and a negative value means that a SGN has more *Gria2* puncta relative to *Gria3* or *Gria4*. The indices were then compared across cochlear regions and sex using separate two-way ANOVAs for each index. Due to the nature of our experimental design, we planned to investigate sex differences of *Gria2*, *3*, and *4* mRNA counts and *Gria3/2* and *Gria4/2* indices in each separate cochlear region. Therefore, we conducted independent samples t-tests for comparisons of these measures between the sexes in each cochlear region *a priori*.

For RT-qPCR data, separate independent samples t-tests were conducted between males and females to compare *flip* and *flop* relative abundances for *Gria2*, *3*, and *4*.

## Results

### Female mice have larger wave I amplitudes than males

**Figure 2:**
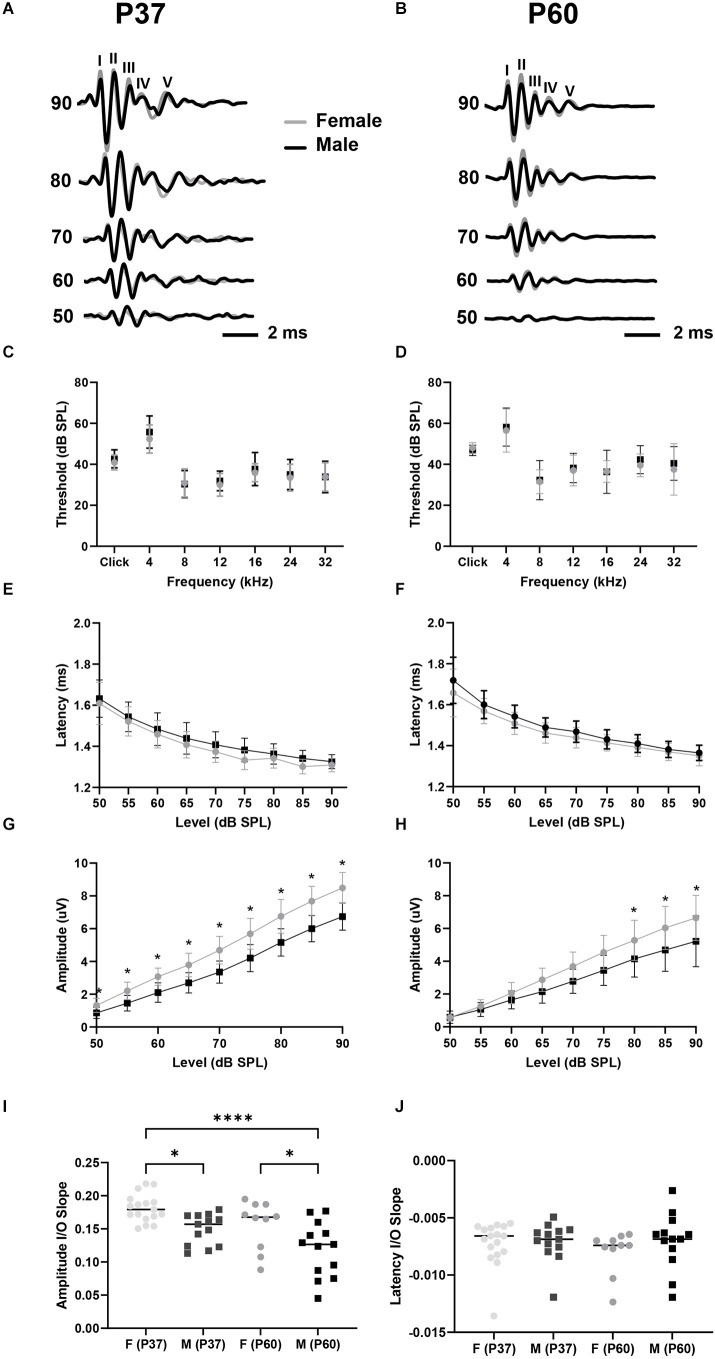
Female mice have larger wave I amplitudes than males. **(A,B)** Representative ABR waveforms evoked with clicks from P37 female (gray line, *n* = 17) and male (black line, *n* = 13) mice **(A)** and P60 female (*n* = 10) and male (*n* = 13) mice **(B)**. Waves—V are labeled. **(C,D)** ABR analysis showed no sex differences in click and pure tones thresholds for P37 **(C)** or P60 **(D)** mice. **(E,F)** There was no sex difference in ABR click wave I latency for P37 **(E)** or P60 **(F)** mice. **(G,H)** Female mice aged P37 have larger click wave I amplitude than male mice at sound intensities equal to and above 50 dB SPL **(G)**, and female mice aged P60 have larger click ABR wave I amplitude at sound intensities equal to and above 80 dB SPL **(H)**. **(I)** Linear slopes of click wave I amplitude as a function of sound level are larger for females aged P37 compared to males aged P37 and P60, and slopes are higher for females aged P60 compared to males aged P60. **(J)** Linear slopes of click wave I latencies do not differ between sexes at any age. Error bars represent ± SD. Lines on **(I,J)** scatterplots denote the median. *p < 0.05, ****p < 0.0001.

We first tested for potential sex differences in ABR threshold and wave I latency and amplitude in our colony of C57BL/6J mice (Figure 2). These parameters are physiological measures of hair cell health (threshold), SGN speed of conduction (wave I latency), and SGN health and synchronous activity (wave I amplitude; see Liberman and Kujawa, 2017; Young et al., 2021). Our data showed that ABR clicks- and tone-thresholds varied by frequency (i.e., broadband click, 4, 8, 12, 16, 24, and 32 kHz pure tones), but not by sex for both P37 and P60 aged cohorts (two-way mixed ANOVA with a main effect of frequency on ABR threshold, no main effect of sex, nor an interaction of sex and frequency; Figures 2C,D; see Table 1 for ANOVA statistics). We next analyzed wave I latencies. Data showed that click wave I latency decreased with increasing sound levels (50–90 dB), but did not vary by sex (two-way mixed ANOVA with main effect for sound level, no main effect of sex, nor an interaction of sex and sound level; Figures 2E,F). There was no difference in slopes of click wave I latency as a function of stimulus level between the sexes of either age cohort (Figure 2J; Table 1). In contrast, we found that click wave I amplitude differed depending on sex and increased with sound levels (50–90 dB) for both age cohorts (two-way mixed ANOVA with main effect of sex, the main effect of sound level, and an interaction between sex and sound level; Table 1). In P37 mice, pairwise comparisons revealed that there was a sex difference at all tested sound levels with a visualized ABR (50–90 dB SPL; *p* < 0.05). In P60 mice, females had significantly larger wave I amplitude than males from 80 to 90 dB SPL (*p* < 0.05; Figures 2G,H). Similarly, analysis of the slope of ABR wave I amplitude as a function of stimulus level revealed that females had higher slopes than males at P37 and P60 (Figure 2I). Wave I amplitudes were also examined for all pure tone stimuli (4, 8, 12, 16, 24, and 32 kHz) for P60 mice. Though not significant, females had slightly higher ABR wave I amplitudes at all frequencies except for 4 kHz (data not shown). Thus, the significant difference for click wave I amplitudes is likely due to a cumulative effect from cochlear regions above 4 kHz. Together, our ABR analysis of sexually mature female and male mice showed no sex differences in click and pure tones thresholds and wave I latency. However, a sex difference in wave I amplitude suggests greater SGN synchrony in females at supra-threshold sound levels, and variability begins to increase at P60, particularly in males.

**Table 1:** Summary of ANOVA statistics.

**Dependent variable**	**Effect**	**F statistic (DF)**	***p*-value**
ABRs			
Threshold			
P37	Sex	1.0 (1, 28)	ns
	Frequency	66.05 (4, 110.5)	<0.0001
	Interaction	0.4 (6, 168)	ns
P60	Sex	0.8 (1, 133)	ns
	Frequency	23.5 (6, 133)	<0.0001
	Interaction	0.2 (6, 133)	ns
Wave I Latency			
P37	Sex	2.0 (1, 28)	ns
	Level	323.2 (1.8, 51.2)	<0.0001
	Interaction	0.83 (8, 224)	ns
P60	Sex	1.8 (1, 21)	ns
	Level	177 (1.5, 30.5)	<0.0001
	Interaction	0.9 (8, 168)	ns
Wave I Amplitude			
P37	Sex	26.0 (1, 28)	<0.0001
	Level	1,061 (2.1, 57.6)	<0.0001
	Interaction	10.2 (8, 224)	<0.0001
P60	Sex	6.2 (1, 21)	0.02
	Level	238.5 (8, 168)	<0.0001
	Interaction	4.4 (8, 168)	<0.0001
AMPAR mRNA			
*Gria*2 Puncta	Sex	9.9 (1, 2957)	0.02
	Region	17.8 (2, 2957)	<0.0001
	Interaction	5.4 (2, 2957)	0.004
*Gria3* Puncta	Sex	9.6 (1, 2122)	0.002
	Region	37.2 (2, 2122)	<0.0001
	Interaction	0.85 (2, 2122)	ns
*Gria4* Puncta	Sex	4.2 (1, 1936)	0.04
	Region	25.8 (2, 1936)	<0.0001
	Interaction	21.7 (2, 1936)	<0.001
*Gria3/2* Index	Sex	7 (1, 2122)	0.008
	Region	3.3 (2, 2122)	0.04
	Interaction	4.5 (2, 2122)	0.01
*Gria4/2* Index	Sex	5.6 (1, 1918)	0.02
	Region	6.2 (2, 1918)	0.002
	Interaction	17.9 (2, 1918)	<0.0001

DF, Degrees of Freedom; ns, statistically non-significant. ANOVA F-statistics and *p*-values for ABR threshold, wave I latency, and wave I amplitude (top); and *Gria2, 3*, and *4* mRNA fluorescent puncta counts and *Gria3/2* and *Gria4/2* indices (bottom).

### Slight differences in *Gria2, Gria3*, and *Gria4* mRNA expression along the rosenthal canal of female and male mice

**Figure 3:**
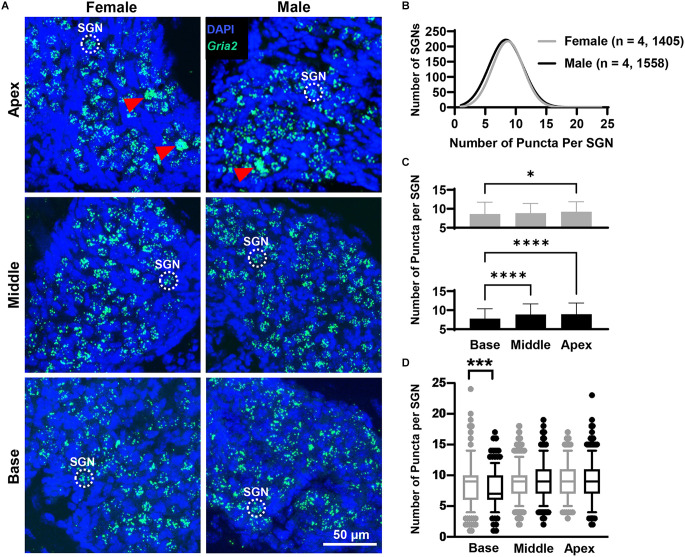
*Gria2* mRNA counts analyzed by cochlear region and sex. **(A)** Representative fluorescent confocal stack of images of *Gria2* (green) and DAPI (blue) in the basal middle, and apical cochlea turns for females and males. Red arrowheads point to SGNs that were excluded due to puncta clustering. **(B)** Histogram representing the distribution of *Gria2* mRNA counts in female (gray line) and male (black line) mice throughout the entire cochlea. Numbers in parentheses indicate the number of mice, followed by the total number of SGNs. **(C)** Bars show the mean *Gria2* mRNA puncta on different cochlea turns on SGNs of female (gray bars, top) and male (black bars, bottom) mice. Error bars represent ± SD. **(D)** Whisker plots of *Gria2* mRNA puncta on different cochlea turns on SGNs of females (gray) and males (black). Females had more *Gria2* mRNA in the basal turn compared to males. Whiskers display the 5 and 95 percentiles. Samples consisted of the total number of SGNs for which *Gria2* puncta were counted in females and males. See Table 2 for sample sizes by region. Independent samples *t*-test, *p < 0.05, ***p < 0.0005, ****p < 0.0001, see “Results” Section for specific *p*-values.

**Figure 4:**
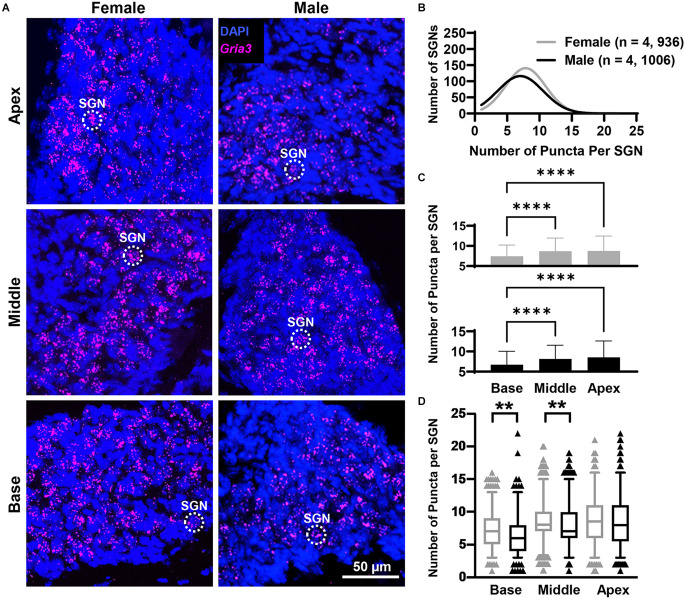
*Gria3* mRNA counts analyzed by cochlear region and sex. **(A)** Representative fluorescent confocal stack of images of *Gria3* (false-colored in magenta) and DAPI (blue) in the base, middle, and apex cochlea turns for females and males. **(B)** Histogram representing the distribution of *GriaA3* mRNA counts in female (gray line) and male (black line) mice throughout the entire cochlea. Numbers in parentheses indicate the number of mice, followed by the total number of SGNs. **(C)** Bars show the mean *Gria3* mRNA puncta on different cochlea turns on SGNs of female (gray bars, top) and male (black bars, bottom) mice. Error bars represent ± SD. **(D)** Whisker plots of *GriaA3* mRNA puncta on different cochlea turns on SGNs of females (gray) and males (black). Females had more *Gria3* mRNA in the basal and middle turns than males. Whiskers display the 5 and 95 percentiles. Samples consisted of the total number of SGNs for which *Gria3* puncta were counted in females and males. See Table 2 for sample sizes by region. Independent samples t-test, **p < 0.01, ****p < 0.0001, see “Results” Section for specific *p*-values.

**Figure 5:**
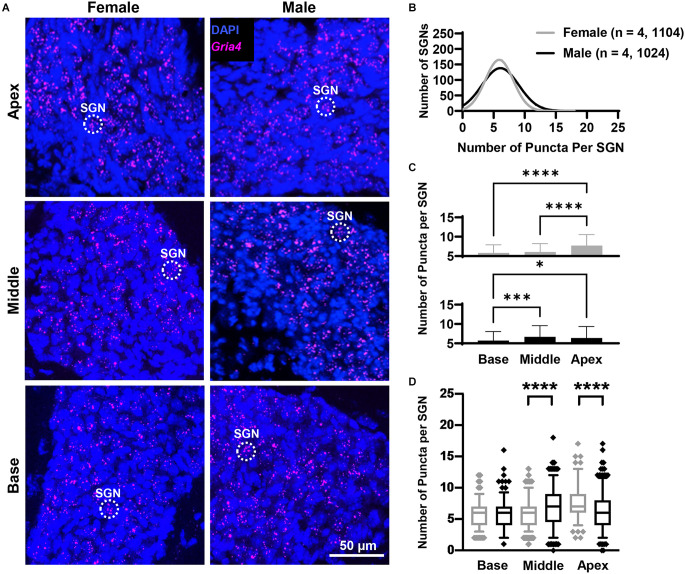
*Gria4* mRNA counts analyzed by cochlear region and sex. **(A)** Representative fluorescent confocal stack of images of *Gria4* (false-colored in magenta) and DAPI (blue) in the base, middle, and apex cochlea turns for females and males. **(B)** Histogram representing the distribution of *Gria4* mRNA counts in female (gray line) and male (black line) mice throughout the entire cochlea. Numbers in parentheses indicate the number of mice, followed by the total number of SGNs. **(C)** Bars show the mean *Gria4* mRNA puncta on different cochlea turns on SGNs of female (gray bars, top) and male (black bars, bottom) mice. Error bars represent ± SD. **(D)** Whisker plots of *Gria4* mRNA puncta on different cochlea turns on SGNs of females (gray) and males (black). Males had more *Gria4* mRNA in the middle turn, while females had more *Gria4* mRNA in the apex. Whiskers display the 5 and 95 percentiles. Samples consisted of the total number of SGNs for which *Gria4* puncta were counted in females and males. See Table 2 for sample sizes by region. Independent samples t-test, *p < 0.05, ***p < 0.0005, ****p < 0.0001, see “Results” Section for specific *p*-values.

In both females and males, puncta of the RNAscope signal for *Gria2*, *Gria3*, and *Gria4* were only observed within and along the Rosenthal canal of the cochlea and around large DAPI nuclei of SGNs (Figures 1, 3A, 4A, 5A). We compared total mRNA levels for *Gria2*, *Gria3*, and *Gria4* between females and males. In addition, for determining whether mRNA levels for *Gria2–4* followed a tonotopic organization and whether there were sex-specific differences in their distribution, we quantified the puncta for each of the genes in SGNs of the basal (high-frequency sounds), middle (middle frequency sounds), and apical (low-frequency sounds) cochlea turns.

#### *Gria2* mRNA counts

Overall, females and males had similar *Gria2* mRNA expression levels (Figure 3B). We found that *Gria2* mRNA levels varied by sex and cochlear region (two-way ANOVA with main effect of sex, main effect of cochlear region, and interaction of sex and cochlear region; Figure 3C; Table 1). In both females and males, *Gria2* mRNA levels were lower in the basal compared with the apical turn (female *p* = 0.02, male *p* < 0.0001). In males, *Gria2* expression levels were also lower in the basal compared with the middle turn (*p* < 0.0001). Pairwise comparisons between females and males at each cochlear region revealed that females had greater *Gria2* mRNA than males at the base, and this sex difference explained 2% of the variance of *Gria2* mRNA in the cochlear base (*p* = 0.0003, η^2^ = 0.02). There was no sex difference in the middle (*p* = 0.8) and apical cochlea turns (*p* = 0.2; Figure 3D; see Table 2 for descriptive statistics).

**Table 2:** Summary of AMPAR mRNA descriptive statistics.

**Dependent variable**	**v**	**Sex (n)**	**Mean (± SD)/SGN**	**Range**
*Gria2* Puncta	Base	M (275)	7.8 (3.1)	1–17
		F (357)	8.6 (2.6)	1–24
	Middle	M (830)	8.9 (2.8)	2–19
		F (813)	8.8 (2.6)	2–18
	Apex	M (453)	8.9 (2.9)	2–23
		F (235)	9.2 (2.6)	3–17
*Gria3* Puncta	Base	M (219)	6.7 (3.4)	1–22
		F (390)	7.4 (2.8)	1–16
	Middle	M (512)	8.1 (3.4)	1–19
		F (466)	8.7 (3.3)	1–20
	Apex	M (293)	8.6 (4.1)	1–22
		F (248)	8.8 (3.7)	1–21
*Gria4* Puncta	Base	M (194)	5.7 (2.4)	1–16
		F (248)	5.8 (2.1)	2–12
	Middle	M (525)	6.7 (2.9)	0–18
		F (547)	6.0 (2.2)	1–13
	Apex	M (287)	6.4 (3.0)	0–17
		F (141)	7.7 (2.8)	2–17
*Gria3/2* Index	Base	M (219)	−0.04 (0.25)	−0.75–0.50
		F (390)	0.01 (0.25)	−0.86–0.64
	Middle	M (512)	−0.02 (0.22)	−0.70–0.60
		F (466)	0.04 (0.23)	−0.80–0.60
	Apex	M (293)	−0.01 (0.27)	−0.75–0.64
		F (248)	−0.03 (0.25)	−0.87–0.70
*Gria4/2* Index	Base	M (194)	−0.15 (0.23)	−0.60–0.70
		F (248)	−0.16 (0.26)	−0.70–0.80
	Middle	M (525)	−0.17 (0.24)	−1.0–0.47
		F (547)	−0.22 (0.22)	−0.80–0.56
	Apex	M (287)	−0.22 (0.25)	−1.0–0.47
		F (123)	−0.08 (0.22)	−0.6–0.38

F, Females; M, Males; SD, Standard Deviation; SGN, Spiral Ganglion Neurons. Means, standard deviations, and ranges of fluorescent puncta counts of *Gria2, 3*, and *4* mRNA (top) and *Gria3/2* and *Gria4/2* indices by cochlear region (base, middle, and apex) for each sex. Sample sizes represent the total number of SGNs for each sex per region from a total of four males and four females.

#### *Gria3* mRNA counts

Overall, females had more *Gria3* mRNA than males (Figure 4B).* Gria*3 mRNA counts were affected by sex and cochlear region (two-way ANOVA with the main effect of sex, and main effect of cochlear region, but no interaction of sex and cochlear region; Figure 4C; Table 1). In females and males, *Gria3* mRNA levels were lower in the basal cochlea turn when compared to the middle turn (*p* < 0.0001 for both comparisons). In pairwise comparisons between males and females at each cochlear region, females had greater *Gria3* mRNA puncta in the basal turn compared to males, explaining 2% of the variance of *Gria3* mRNA in the base (*p* = 0.009, η^2^ = 0.02). Females also had more *Gria3* mRNA in the middle turn compared to males, with sex explaining 1% of the variance in the middle (*p* = 0.01, η^2^ = 0.01). There was no sex difference in the apex (*p* = 0.6; Figure 4D; Table 2).

#### *Gria4* mRNA counts

Overall, females and males had similar *Gria4* mRNA expression levels (Figure 5B).* Gria4* mRNA levels similarly varied by sex and cochlear region (two-way ANOVA with main effect of sex, cochlear region, and interaction of sex and cochlear region; Figure 5C; Table 1). In females, *Gria4* mRNA levels were similar between the base and middle cochlea turns (*p* = 0.37) and only the apex had significantly higher levels of expression for *Gria4* (*p* < 0.0001). In males, *Gria4* mRNA levels were similar between the middle and apex (*p* = 0.28), but lower in the base (*p* = 0.0001 and *p* = 0.03, respectively). Pairwise comparisons showed that males had greater amounts of *Gria4* in the middle turn compared to females and that sex explains 2% of the variance in *Gria4* levels (*p* < 0.0001, η^2^ = 0.02), while females had more *Gria4* at the apex compared to males and sex accounts for 5% of the variance in *Gria4* mRNA in this cochlea region (*p* < 0.0001, η^2^ = 0.05; Figure 5D; Table 2).

Our analysis shows that in both sexes, the total *Gria2*, *3*, and *4* mRNA levels increased from the basal to the apical cochlea turns following a tonotopic gradient from high to low frequencies. Additionally, when compared to males, data showed that females had more total mRNA levels of *Gria2* and *Gria3* in the basal turn, more *Gria3* in the middle turn, and more *Gria4* in the apical turn. In contrast, males had more total *Gria4* mRNA in the middle cochlea turn. Of note is that sex accounts for a small amount of variability in total mRNA expression of all three subunits (1%–5%) between male and female mice.

### Subpopulations of SGNs along the rosenthal canal based on *Gria2, Gria3*, and *Gria4* expression levels

**Figure 6:**
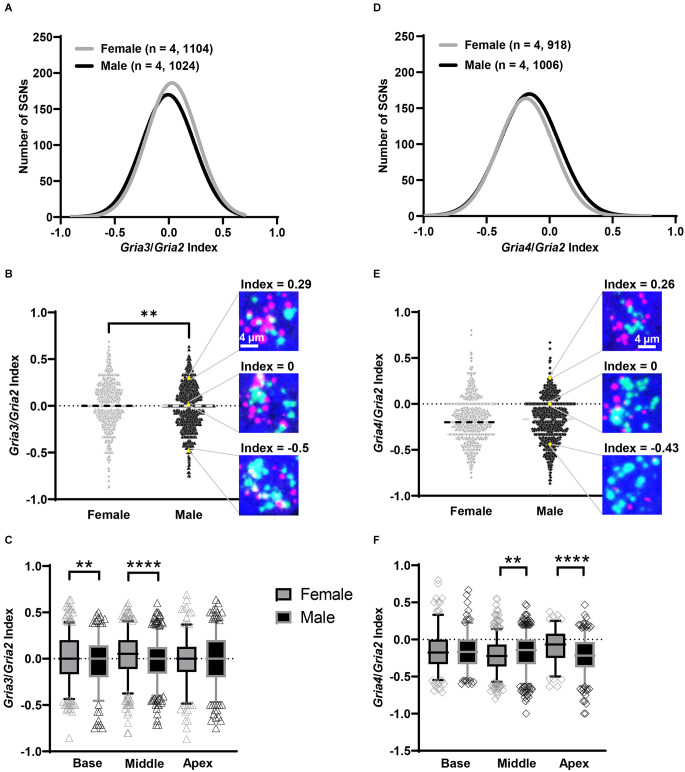
SGN *Gria3/Gria2* and *Gria4/Gria2* indices vary by sex. **(A–C)** Subpopulations of SGNs based on the *Gria3/Gria2* index value. **(A)** Histogram showing the total distribution of SGN *GriaA3/Gria2* indices for females (gray line) and males (black line). Numbers in parentheses indicate the number of mice, followed by the total number of SGNs. **(B)** Violin plots of the index values for *Gria3/Gria2* on each SGN for the whole cochlea. To the right are representative images of SGNs in a male with different index values based on *Gria3* (magenta) vs. *Gria2* (green) counts: *Gria3* > *Gria2* (top, positive index value), *Gria3* = *Gria2* (middle, index value of 0), and *Gria3* < *Gria2* (bottom, negative index value). The position of each example SGN within the violin plot is represented with a yellow triangle. The dashed line on each violin plot represents the median. **(C)** Whisker plots show the index values for *Gria3/Gria2* on SGNs of the basal, middle, and apical cochlea turns. Females (gray symbols) had higher average *Gria3/Gria2* index values in the base and middle compared to males (black symbols). Whiskers display the 5 and 95 percentiles. **(D–F)** Subpopulations of SGNs based on the *Gria4/Gria2* index value. **(D)** Histogram showing the total distribution of SGN *Gria4/Gria2* indices for females (gray line) and males (black line). Numbers in parentheses indicate the number of mice, followed by the total number of SGNs. **(E)** Violin plots of the index values for *Gria4/Gria2* on each SGN for the whole cochlea. To the right are representative images of SGNs in a male with different index values based on *Gria4* (magenta) vs. *Gria2* (green) counts: *Gria4* > *Gria2* (top, positive index value), *Gria4* = *Gria2* (middle, index value of 0), *Gria4* < *Gria2* (bottom, negative index value). The position of each example SGN within the violin plot is represented with a yellow diamond. The dashed line on each violin plot represents the median. **(F)** Whisker plots show the index values for *Gria4/Gria2* on SGNs of the base, middle, and apex cochlea turns. Males (black symbols) had on average higher *Gria4/Gria2* index values in the middle, while females (gray symbols) had a higher average *Gria4/Gria2* index in the apex. Whiskers display the 5 and 95 percentiles. Unpaired *t*-test, **p < 0.01, ****p < 0.0002.

In our RNAscope procedure, each SGN had mRNA expression for two genes, either *Gria2* and *Gria3* or *Gria2* and *Gria4*. Our initial semiquantitative analysis of the puncta showed in both female and male mice that SGNs differed in the ratios of the *Gria3/Gria2* and *Gria4/Gria2* puncta (Figure 1). For example, some SGNs had more *Gria3* than *Gria2* puncta and *vice versa*; other SGNs had roughly equal amounts of *Gria3* and *Gria2* puncta. We used an index calculation to classify the putative existence of a heterogenous population of SGNs based on *Gria2*, *3*, and *4* mRNA levels (Balaram et al., 2019). The *Gria3/Gria2* and the *Gria4/Gria2* index calculations revealed a gradient of SGNs based on these indices in males and females throughout the cochlea (Figure 6; see Section “Materials and Methods” for details). We found a population of SGNs with a positive index value (> 0), meaning that these neurons had higher mRNA levels for *Gria3* or *Gria4* than *Gria2*. Data also showed another population of SGNs with an index value of 0, meaning that they had equal mRNA levels for each of the paired genes analyzed. Finally, data showed a third population of SGNs with a negative index value (< 0), which indicates that *Gria2* mRNA is higher than *Gria3* or *Gria4*. We found that the percentage of each population of SGN differed along the cochlea turns and by sex.

#### *Gria3/2* index

When analyzing the entire cochlea (base, middle, and apex), 48% of the female SGNs had a positive *Gria3/2* index value compared to 43% in males. The percent of SGNs with an index value of zero was 11% in both females and males, while those with a negative index value were 41% in females and 46% in males. While we are interested in sex differences in the proportion of these three “classes” of SGN, we chose to analyze the indices as continuous variables because these boundaries for classification are somewhat arbitrary. Indeed, an SGN with a slightly positive or negative index value is likely similar to an SGN with an index value of zero in terms of AMPAR composition. Like mRNA counts, *Gria3/2* indices varied by sex and cochlear region (two-way ANOVA with the main effect of sex, main effect of cochlear region, and an interaction of sex and cochlear region; Table 1). Throughout the entire cochlea, on average females had SGNs with more *Gria3* relative to *Gria2* (mean *Gria3/2 index* = 0.02 ± 0.24 SD) compared to males (mean *Gria3/2* index = −0.02 ± 0.24 SD; Figures 6A,B). In pairwise comparisons, females had a higher average *Gria3/2* index in the basal turn compared to males (*p* = 0.02, η^2^ = 0.01) and in the middle turn (*p* < 0.001, η^2^ = 0.02). There was no sex difference in the apical cochlea turn (*p* = 0.41; Figure 6C; Table 2).

#### *Gria4/2* index

The *Gria4/2* index calculations revealed that the percent of SGNs with positive, zero, and negative indices were closer between females and males. The percentage of SGN with a positive index was 17% in females and 19% in males. The percentage of SGN with an index of zero was 8% and 8.5% in females and males, respectively. Finally, the percentage of SGN with a negative index in females was 75% and 72.5% in males. However, *Gria4/2* indices in SGNs varied by sex and cochlear region (two-way ANOVA with main effect of sex, cochlear region, and an interaction of sex and cochlear region; Table 1). Throughout the entire cochlea, though there was an effect of sex in the ANOVA, on average males and females had a similar *Gria4/2* index (male mean = −0.182 ± 0.24 SD, female mean = −0.181 ± 0.23 SD; Figures 6D,E). In pairwise comparisons, males had a higher average *Gria4/2* index compared to females in the middle turn (*p* = 0.005, η^2^ = 0.01), while females had a higher average *Gria4/2* index than males in the apical turn (*p* < 0.0001, η^2^ = 0.06). There was no sex difference in the cochlear base (*p* = 0.71; Figure 6F; Table 2). However, it is important to note that despite these sex differences, which are relatively small, on average, females and males had SGNs with less *Gria4* relative to *Gria2* throughout the entire cochlea (Figure 6).

In summary, our data suggest that there is a large population of SGNs with more *Gria3* relative to *Gria2* (>40% of all SGNs analyzed) and a smaller population of SGNs with more *Gria4* relative to *Gria2* (<20% of all SGNs analyzed) regardless of sex. When comparing the sexes, females had more SGNs with positive *Gria3/2* indices than males in the cochlear base and middle. In *Gria4/2* index sex comparisons, males had higher average *Gria4/2* index values in the middle cochlea, and females had higher average *GriaA4/2* values in the apex. Though significant, sex has a small effect on the variability of the *Gria3/2* and *Gria4/2* indices (1%–6%).

### Sex differences in *Gria3* and *Gria4* flip and flop splice variants in the mouse inner ear

**Figure 7:**
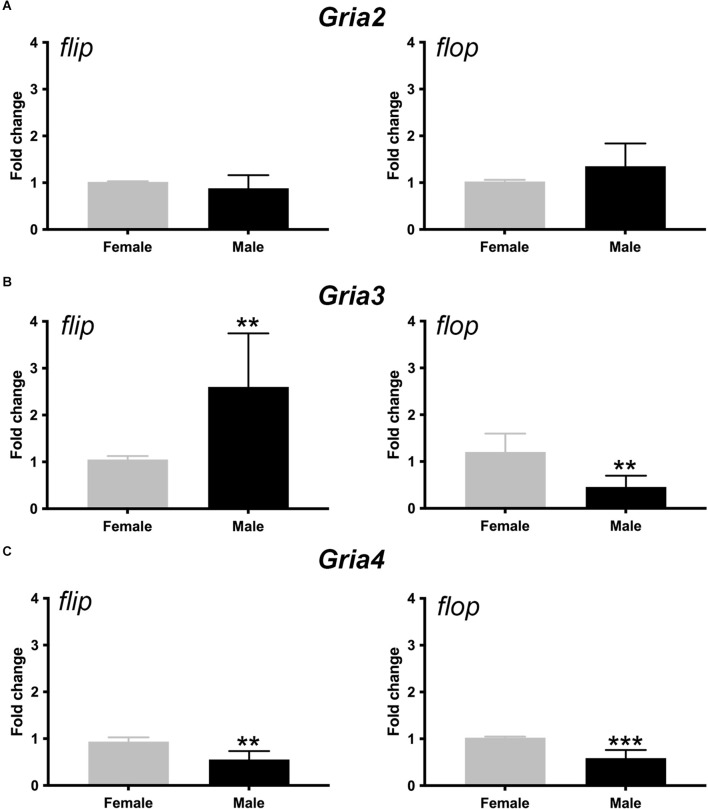
Sex differences in the expression of AMPAR *flip* and *flop* splice variants in the cochlea. **(A–C)** Bars show the fold change (±SD) in mRNA for *Gria2* **(A)**, *v3* **(B)**, and *Gria4* **(C)** *flip* and *flop* splice variants in male (black) when compared to female (gray) cochleae. Independent samples *t*-test, **p < 0.05, ***p < 0.0005, see “Results” Section for specific *p*-values.

AMPAR subunit composition determines channel permeability to calcium and sodium, and the gating kinetics of the channel is determined by the *flip-and-flop* splice variants (Mosbacher et al., 1994). Thus, we next determined *Gria2*, *3*, and 4 *flip* and *flop* splice variant mRNA levels in the female and male cochlea with RT-qPCR. Our data showed that the relative abundances of *Gria2 flip* and *flop* mRNA were not different between females and males (*t* = 1.198, *p* = 0.26 and *t* = 1.631, *p* = 0.13, respectively). However, males had roughly 2.5-fold more *Gria3* *flip* mRNA than females, whereas females had roughly 2-fold more *Gria3* *flop* mRNA than males (*t* = 3.32, *p* = 0.007, and *t* = 3.986, *p* = 0.002, respectively). Additionally, females had roughly 2-fold more *Gria4* *flip* and *flop* compared to males (*t* = 4.27, *p* = 0.002 and *t* = 5.56, *p* = 0.0005, respectively; Figure 7). These data suggest that females have more post-transcriptional processing of *Gria3* into the faster *flop* isoform and *Gria4* into the slower *flip* and faster *flop* isoform than males throughout the entire cochlea. In contrast, males had more of the slower gating *Gria3* *flip* isoform. We note that these results were obtained from the entire inner ear that included SGN and vestibular neurons.

## Discussion

Supra-threshold ABR wave I amplitudes measure SGN synchrony which depends partly on the channel kinetics of excitatory postsynaptic AMPA-type glutamate receptors. Human and rodent studies (this study; Kjær, 1979, 1980; Wharton and Church, 1990; Balogová et al., 2018; Milon et al., 2018) have shown that ABR wave I amplitudes are higher in females. While the potential molecular mechanisms of this sex difference are unknown, our data show that sex differences in AMPAR subunit expression and their differences in alternative splicing (*flip*-and-*flop*) may contribute to greater SGN synchrony and as a result to higher ABR wave I amplitude in females.

### AMPAR subunit composition contributes to synapses speed

Relative levels of each AMPAR subunit (GluA2, 3, and 4) are significant contributors to synaptic speed in SGNs because they affect the channel kinetics of AMPARs differently. AMPARs that lack GluA2 are calcium permeable and are known to have extremely fast channel kinetics (Cull-Candy et al., 2006; Traynelis et al., 2010). For example, cochlear nucleus principal neurons that receive excitatory synaptic input from auditory nerve fibers where fast synaptic responses are essential for reliably encoding auditory information, AMPARs are mainly composed of GluA3 and GluA4 subunits (Rubio and Wenthold, 1997; Wang et al., 1998; Gardner et al., 1999, 2001; Rubio et al., 2017). While many of these studies focused on mature AMPAR subunit proteins at the synapse, importantly others have shown that subunit mRNA levels correlate with channel kinetics. Indeed, studies in the CNS have shown that faster AMPAR gating kinetics correlate with *Gria4* mRNA levels, but not with *Gria2* mRNA levels (Geiger et al., 1995). Furthermore, relative levels of alternatively spliced AMPAR subunit *flip* and *flop* isoforms more accurately describe each subunit’s contribution to gating kinetics (Sommer et al., 1990; Mosbacher et al., 1994; Lambolez et al., 1996; but see Angulo et al., 1997). In fact, in cells expressing recombinant AMPARs, homomeric AMPAR channels containing *Gria3* and *Gria4* *flip* are 3.4 and 4x slower gating, respectively, than those containing the *flop* isoforms of each (Mosbacher et al., 1994). The *Gria2* isoform has a weaker effect on AMPAR desensitization, though *Gria2 flop* increases the speed of desensitization relative to the *flip* isoform in both homomeric *Gria2* and heteromeric *Gria2/3* and *Gria2/4* channels (Mosbacher et al., 1994; Koike et al., 2000). Thus, SGNs enriched with *flop* isoforms of the GluA3 or GluA4 subunits will always have AMPAR channels with faster kinetics and therefore faster synaptic responses than SGNs enriched with the *flip* isoform. Based on this prior evidence, we propose that SGNs enriched in *Gria3* and/or *Gria4* mRNA, especially in the *flop* isoform, will likely have increased temporal precision in response to sound, thus contributing to greater synchrony and higher ABR wave I amplitudes.

### SGNs are heterogenous in relative mRNA expression levels of *Gria2, 3*, and *4*

Our analysis of AMPAR subunit mRNA showed that the relative expression of the three AMPAR subunits (*Gria2, 3*, and *4*) are not equal in individual SGNs, as evidenced by the differences in *Gria3/2* and *Gria4/2* indices. This result indicates the existence of a heterogenous population of SGN that could differ in AMPAR channel kinetics and permeability to calcium and sodium. For example, AMPARs lacking GluA2 are calcium-permeable and recently have been proposed as mediators of SGN excitotoxicity (Sebe et al., 2017; Hu et al., 2020). Thus, SGNs with lower levels of GluA2 relative to GluA3 and GluA4 will not only likely have faster synaptic responses once depolarized but will be more vulnerable to excitotoxicity due to AMPARs with higher permeability to calcium and sodium (Cull-Candy et al., 2006; Traynelis et al., 2010; Sebe et al., 2017; Hu et al., 2020). Interestingly, women are better protected from sensorineural hearing loss than men (Cruickshanks et al., 1998; Agrawal et al., 2008), and estrogen has been shown to have oto-protective effects in mice (Meltser et al., 2008; Simonoska et al., 2009; Williamson et al., 2019). Thus, the mechanism underlying sex differences in auditory processing may differ from the protective mechanism of estrogens. Whether SGNs can also be classified by differences in the AMPAR subunits’ *flip/flop* ratio, is still undetermined, because our analysis was only performed from whole inner ear samples rather than on single SGNs. Future studies that induce, for example, excitotoxicity in SGNs coupled with RNAscope, electrophysiology, and single-cell RT-qPCR for *Gria2*, *3*, and *4* and their splice variants (*flip* and *flop*) will increase our understanding of whether these different SGNs differ in their fast AMPAR synaptic responses and gating, and calcium permeability.

### Sex differences in AMPAR subunit mRNA

Since females have higher ABR wave I amplitude (Figure 2), we hypothesized that they would have more overall AMPAR mRNA that could contribute to fast channel kinetics compared to males. Three findings from our mRNA quantification experiments suggest that *Gria3* is the most likely AMPAR subunit to potentially mediate increased SGN synchrony in females. For one, females have more overall *Gria3* mRNA than males (Figure 4). Second, females have a significantly higher *Gria3/2* index overall than males, meaning the gradient of SGNs with *Gria3* relative to *Gria2* is shifted higher in females (Figures 6A–C). Last, *Gria3* was post-transcriptionally processed into the *flop* isoform to a greater level in females than males who had more *Gria3* *flip* (Figure 7). Thus, SGNs of the female cochlea have more overall *Gria3* mRNA in the *flop* isoform, which was previously shown to lead to faster AMPAR gating kinetics than *Gria3* *flip* whether it was dimerized to *Gria2* *flip* or *flop* (Mosbacher et al., 1994). Also, more *Gria3* relative to *Gria2* suggests that females may have more heteromeric AMPARS containing *Gria3* and *Gria4*, which are known to comprise AMPARs in fast excitatory synapses in the auditory CNS (Rubio and Wenthold, 1997; Gardner et al., 2001; Rubio et al., 2017). Our study shows that SGNs of female and male mice differ in their mRNA expression levels for *Gria2, 3*, and *4* AMPAR subunits, thus potentially contributing to our understanding of basic molecular mechanisms of sex differences in normal hearing mice.

### Sex differences in AMPAR subunit composition: implications for glutamatergic synapse-induced excitotoxicity

Glutamate-induced calcium excitotoxicity *via* AMPARs does not only occur in the cochlea (Sebe et al., 2017; Hu et al., 2020), but also in other brain regions. In fact, calcium-permeable (GluA2 lacking) AMPARs are implicated in neuronal cell death and in neurological disorders in the CNS (Liu and Zukin, 2007; Guo and Ma, 2021). The sex differences in AMPAR subunits gene expression we describe here may not be specific to SGNs in the inner ear but present in other glutamatergic neurons in the CNS. For example, in human hippocampal neurons, schizophrenia patients had less overall *Gria2* than controls (Eastwood et al., 1997), suggesting that differences in AMPAR subunit transcription could contribute to the disease. Interestingly, women and men differ in presenting schizophrenic symptoms and response to antipsychotic medication. There is some evidence that both sex hormones and sex chromosomes are related to schizophrenia in women and men (Li et al., 2016). Thus, probing sex differences in AMPAR subunit mRNA and the alternatively spliced *flip* and *flop* isoforms may aid in understanding sex differences in neurological disorders involving glutamatergic signaling through AMPARs.

### Proximate mechanisms of biological sex on *Gria3* expression

The mechanism for the sex difference in AMPAR subunit mRNA expression described in this study is still unknown. Estrogen receptors (ER) and androgen receptors (AR) are both transcription factors (Levin and Hammes, 2016), and therefore estrogens and androgens could regulate the expression of AMPAR subunits either directly or indirectly *via* the recruitment of other transcription factors. Evidence that ERα and ERβ are found in SGNs (Stenberg et al., 1999; Meltser et al., 2008; Shuster et al., 2021) together with data from knockout mouse models have demonstrated that ERβ is important for SGN health (Simonoska et al., 2009). On the other hand, while the *Gria2* and *Gria4* genes are located on autosomes, the *Gria3* gene is located on the X chromosome (Mahadevaiah et al., 2009). Therefore, it is also possible that females have more *Gria3* mRNA due to incomplete X-inactivation in some or all SGNs leading to increased gene dosage (Carrel and Willard, 2005; Berletch et al., 2011). This would suggest that this sex difference is not due to hormonal differences but rather intrinsic biological sex due to the XX/XY sex-determination system. It is essential to study potential anatomical and molecular sex differences in the nervous system to better tailor treatment options for optimal auditory processing in both sexes.

## Data availability statement

The raw data supporting the conclusions of this article will be made available by the authors, without undue reservation.

## Ethics statement

The animal study was reviewed and approved by University of Pittsburgh IACUC.

## Author contributions

NL, SM, H-MC, IP, and MR: performed research and analyzed data. NL: writing—original draft. MR: designed research, writing—review and editing. The authors have read and have abided by the statement of ethical standards for manuscripts submitted to Neuroscience. All authors contributed to the article and approved the submitted version.

## Footnotes

^1^ https://imagej.nih.gov/ij/
